# Maternal care shapes an aposematic display and provides lifelong protection against predators

**DOI:** 10.1093/beheco/araf116

**Published:** 2025-09-29

**Authors:** C Lindstedt, G Boncoraglio, S C Cotter, J D J Gilbert, R M Kilner

**Affiliations:** Department of Forest Sciences, University of Helsinki, Latokartanonkaari 7, P.O. Box 27, 00014 Helsinki, Finland; Department of Zoology, University of Cambridge, Downing St, CB2 3EJ, Cambridge, United Kingdom; Department of Zoology, University of Cambridge, Downing St, CB2 3EJ, Cambridge, United Kingdom; School of Natural Sciences, University of Lincoln, Brayford Pool, LN6 7TS, Lincoln, United Kingdom; Department of Zoology, University of Cambridge, Downing St, CB2 3EJ, Cambridge, United Kingdom; School of Life and Environmental Sciences, The University of Hull, Cottingham Road, HU6 7RX, Hull, United Kingdom; Department of Zoology, University of Cambridge, Downing St, CB2 3EJ, Cambridge, United Kingdom

**Keywords:** chemical defense, developmental constraints, insects, parental care, signal evolution, warning coloration

## Abstract

Parental care can improve early offspring survival against predators by providing protection and resources. However, we have little knowledge of how its effects shape predator-prey interactions later in life. We investigated this with the burying beetle *Nicrophorus vespilloides* which provides care for offspring and carries warning coloration to advertise its chemical defenses to predators. Warning displays by prey are selected by predators for uniformity and to reliably advertise the extent to which individuals are chemically defended. We investigated whether the strength of the correlation between the conspicuousness of the warning display and the potency of the chemical defenses depends on levels of care received during development by manipulating the level of maternal care received by larvae and tracking the effects into adulthood. We found that individuals that received limited care, developed into smaller adults with less conspicuous warning displays. The correlation between the visual display and the chemical defense was also weaker when broods received little care as larvae. We conclude that maternal care received by burying beetles modulates the information content of aposematic defense: less care makes signals less reliable. Our results further suggest that the prey's social environment could constrain the response to selection from predators on warning signal reliability.

## Teaser

Offspring that receive reduced parental care produce less reliable warning displays. We demonstrate that for carrion beetle adults, those that received little care as larvae from their mothers exhibited a weak correlation between the salience of their warning display and their level of chemical defense. This suggests that their warning displays are less trustworthy, and therefore potentially ineffective against predators.

## Introduction

The central question in aposematism theory is to understand why selection by predators can result in a continuum of responses in warning signal expression: from uniformity within and among species (Müllerian mimicry) at one end, through to extensive local and geographic variation in signal expression at the other (Briolat et al. 2019). To answer this question, research has traditionally focused on determining the evolutionary outcomes of predator-prey interactions, the demographic history of the populations or the selective constraints imposed by the ecological or physiological condition of both the prey and the predator (Briolat et al. 2019). Recently it has become evident that the predator's social environment additionally influences warning signal evolution, by changing patterns of selection on prey. For example, social information received from other conspecifics affects how predators choose their prey and what kind of prey they should avoid (Thorogood et al. 2018). However, much less is known about how the social environment of the aposematic prey species modulates their response to selection by predators (but see eg (Naisbit et al. 2001; Maan and Cummings 2009) for effects of sexual selection and mate choice on dual functions of warning displays). Yet many aposematic species such as social wasps and bees (Chatelain et al. 2023), gregarious Lepidopteran larvae and sawflies (eg Wang et al. 2021; Lindstedt et al. 2022), as well as aposematic vertebrates (eg Brown et al. 2010; Stynoski et al. 2014b), inhabit a rich social environment of conspecifics as a result of their gregarious lifestyle, or through parental care, or eusociality.

Here we address this neglected issue by analyzing how the level of maternal care received during development influences the expression of warning signal traits involved in predator-prey interactions later in life, during adulthood. One major hypothesis for the emergence of parental care is its role in helping offspring overcome various abiotic and biotic hazards (Clutton-Brock 1991; Royle et al. 2012) such as predation risk (Giesing et al. 2011; Kozak and Boughman 2012), thermoregulation (Grew et al. 2019; Malik et al. 2025), or microbial competition (Rozen et al. 2008). The level of care supplied by parents is well known to be influenced by diverse intraspecific interactions including sexual conflict between parents, conflict between parents and offspring and competition and cooperation between offspring over resource division (Moore et al. 1997; Parker et al. 2002; Lock et al. 2004; Schrader et al. 2015; Jarrett et al. 2017). Since interactions within the family play out during development, they can have a major, non-genetic role in determining an individual's phenotype (Lock et al. 2004; Wade et al. 2010; Kilner et al. 2015; Jarrett et al. 2017; Van Meyel and Meunier 2022). Importantly, these effects can persist into adulthood and can shape an individual's fitness throughout its lifespan (eg Kozak and Boughman 2012; Kilner et al. 2015; Van Meyel and Meunier 2022). In aposematic species, previous studies have shown that parental care protects juveniles against predators by chemically defending offspring in diverse ways (Pavelka et al. 1977; Nishida 2002; Williams et al. 2011; Stynoski et al. 2014a, 2014b). However, it is unclear whether the social environment created by conspecifics can influence the extent of phenotypic variation in warning displays that is then exposed to selection due to predation (Bleakley and Brodie 2009).

We investigated this problem with the aposematic burying beetle (Figure S1), *Nicrophorus vespilloides*, an insect that exhibits biparental care (Lindstedt et al. 2017). Parents breed on the carcass of a small vertebrate, like a mouse or a songbird, which they convert into an edible nest for their young (Eggert et al. 1998). They bury it below ground, shave off the fur or feathers, roll the flesh into a ball and cover it in antimicrobial fluids (Cotter and Kilner 2010). After the larvae hatch, the parents stay with their young to defend the carrion and their offspring from attackers, and they also feed their larvae via oral trophallaxis (Eggert et al. 1998). Parental care is facultative in this species; there is considerable natural variation in the duration of post-hatching care among families (Parker et al. 2015; De Gasperin et al. 2016; Jarrett et al. 2018). Some broods receive no post-hatching care at all, but some larvae can nevertheless survive (Jarrett et al. 2017).

Adult burying beetles bear orange and black coloration (Figure S1), which is used to communicate their chemical defenses to predators (Lindstedt et al. 2017). When handled, they produce a droplet of foul-smelling fluid from their abdomen (Lane and Rothschild 1965; Degenkolb et al. 2011; Lindstedt et al. 2017). As production of responsive chemical defenses is often costly (Higginson et al. 2011; Lindstedt et al. 2018, 2020a), these kind of toxic defenses of an aposematic individual are considered to contribute to a “public good” by educating predators to avoid prey bearing a similar signal in future encounters (Speed et al. 2012). Through this public goods system of education, predators are theoretically predicted to select for uniformity and reliability in warning displays (Mallet and Barton 1989; Kapan 2001; Beatty et al. 2004). Furthermore, greater levels of toxicity (Leimar et al. 1986; Skelhorn and Rowe 2006) and pronounced signal patterns (Lindstedt et al. 2008; Mappes et al. 2014) are expected to evolve under directional, correlated selection by predators (White and Umbers 2021), as all these traits enhance the avoidance learning rate of predators by making warning displays memorable and easy to recognize. Based on the previous studies, we can assume that large orange pattern size (Lindstedt et al. 2008; Mappes et al. 2014) and its higher salience in terms of color richness (ie saturation, eg, from yellowish orange to deeper orange-to-red pigmentation) (Lindstedt et al. 2011a; Nokelainen et al. 2012; María Arenas et al. 2015) would indicate more effective warning signals against predators in the *N. vespilloides*. In addition, individuals that are able to deploy higher volumes and more potent defensive secretions should be better defended against predators (Lindstedt et al. 2017).

In this study, our first aim was to ask if reduced maternal care received during the larval stage can influence the expression of warning signals and their associated chemical defenses in adulthood. We predicted that the supply of care may independently fund both the cost of producing toxic chemical defenses and the pigmentation used in the warning signal. Individuals under full care should have better access to resources eg via provisioning of food during the larval stage (Pilakouta et al. 2018; Burdfield-Steel et al. 2019). As a result, their warning signals should be larger and more salient, and chemical defenses should also be more repellent as they can allocate more resources for synthesizing pigments and defensive compounds for their warning display.

Our second aim was to test if these different components of the aposematic display are correlated and if the supply of care influences the strength of this correlation, thereby causing variation in the extent to which the signal accurately indicates the potency of an insect's chemical defenses (Speed et al. 2010). A scenario like this might arise via a resource-based tradeoff if both the production of the signal and defensive chemicals rely on the same resource supplied by parents (Blount et al. 2009, 2012; Holen and Svennungsen 2012). This could be due to a physiological link, for example if the antioxidants required for both bright pigmentation and protection of tissues against autotoxicity are limited and supplied by parents. Variation in the availability of antioxidants in the diet could then result in signals and defense evolving positively or negatively in relation to each other (Blount et al. 2009, 2012). Alternatively, signal and defense traits could be genetically correlated due to pleiotropy and/or gene linkage, and the strength of covariation expressed in an individual's phenotype could be shaped by limited access to the energy required to produce these traits to their full potential (Hoffmann and Merilä 1999). To test whether the social environment can alter the covariation of signal salience and levels of chemical defense within and among broods, we measured the family-level correlations between these traits across treatment groups with varying amounts of maternal care.

By addressing these two aims, we determined how the supply of care received in early life affects the expression of the aposematic display in adult burying beetles and modulates its information content.

## Material and methods

### *N. vespilloides* colony

We used the same outbred laboratory population of burying beetles established in 2005 at Cambridge University as described in (Lindstedt et al. 2017). Individual adults were housed in moist soil in plastic boxes (12 × 8 × 2 cm) and fed minced beef twice weekly. Boxes were kept at a constant temperature of 21 °C on a 16 h:8 h light:dark cycle. For breeding, unrelated beetles were paired in plastic boxes (17 × 12 × 6 cm) half-filled with moist soil. Each pair was provided with a freshly thawed mouse carcass (21.94 ± 0.33 SE g, range 15 to 35 g) and the breeding box placed in a dark cupboard to simulate underground conditions. Larvae dispersed from the carcass ca. 8 days after pairing and eclosed as adults ∼3 weeks later. Sexual maturity was reached 1 to 2 weeks after eclosion.

### Experimental procedure

All treatments were run between July 2010 and September 2011. We set up 100 boxes, each with a carcass and a pair of adults. All males were removed from the boxes ∼53 h after pairing, ie after egg laying but before hatching had commenced. Broods of the widowed females were haphazardly divided among the following four treatments: “0 h” maternal care: mothers removed at hatching; “8 h” maternal care: mothers removed from the boxes 8 h after the larvae hatched; “24 h” maternal care: mothers removed from the boxes 24 h after the larvae hatched; “>48 h” maternal care: mothers retained until larvae dispersed from the carcass to pupate. Larvae typically hatched 71.28 h ± 1.47 SE after pairing (Boncoraglio and Kilner 2012) and therefore boxes were checked every ca. 5 h from 55 to 96 h after pairing to determine the time of hatching. When larvae dispersed, eight days after pairing their parents, they were transferred to separate boxes, to pupate. In total, 97 pairs bred successfully, yielding 24 to 25 families per treatment.

At eclosion, when individuals had developed the typical black and orange coloration, their chemical defenses were measured as described in (Lindstedt et al. 2017). To quantify the chemical defense, we held each beetle and gently tapped the abdomen from the ventral side. We collected the resulting droplet of fluid in a capillary tube, and measured the quantity produced. We aimed to collect samples from five females and five males per family, when the number of offspring per family was sufficiently large. Altogether we sampled 2 to 10 individuals per family (mean = 8.37, SD = 2.04) yielding 812 samples in total. After collecting these samples, individuals were sexed, weighed and stored in a freezer at −20 ° C for color analysis.

To estimate variation in the toxicity of the defensive secretion among parental care treatments, we conducted standard bioassays with ants (*Formica spp*) similar to those described previously (Lindstedt et al. 2017). The burying beetle's aposematic display is likely to have been selected by visual predators, such as scavenging birds (see (Lindstedt et al. 2017), and references therein). Nevertheless, ants and burying beetles are also rivals for carrion and the burying beetle's chemical defenses are likely to deployed against ants (Scott et al. 1987). Additionally, the defensive fluid of burying beetles has a very high pH and it contains several defensive compounds that are known to be deterrent against invertebrate and vertebrate predators (Degenkolb et al. 2011; Lindstedt et al. 2017). Furthermore, for the many key compounds in insects' chemical defense (eg terpenes, alkaloids, cardenolides, iridoid glycosides), the repellence of chemical defenses against ants often correlates with the repellence against avian predators ([Sillén-Tullberg 1990; Lindstedt et al. 2006, 2008, 2011b, 2022; Conner 2009; Reudler et al. 2015], but see [Rojas et al. 2017]). This means the extent to which ants find the chemical defenses of the burying beetles aversive provides a good biomarker of this fluid's toxic potency also against vertebrate predators such as birds (Lindstedt et al. 2017).

We tested the effectiveness of the anal exudate in deterring wood ants (*Formica* spp.) by offering them either a 10% exudate solution (10% anal exudate/90% sugar water) or a 10% control solution (10% water/90% sugar water). We know from our previous work that this concentration of exudates is sufficiently potent to change ant behavior (Lindstedt et al. 2017). The samples we collected to estimate the volume of the fluid produced by individual beetles were pooled together into Eppendorf tubes, by maternal care treatment, and by sex (ie we had one tube for each sex, per maternal care treatment). These pooled samples were stored in a freezer (at −20 °C), until they were used for bioassays. For testing, the samples were thawed and then diluted with a 20% sugar solution (20% sugar, 80% water) to make the solution potentially attractive to ants.

We put 10 l of both exudate and control solutions close to each other (<2 cm) on a transparent, sterilized plastic circle (4 cm in diameter) and offered it to the ants. We repeated the assay three times per nest on three different ant trails, changing the sequence in which the two treatments were presented between repetitions. Replicating each treatment on each ant trail within a nest takes into account the potential variation in ant traffic. Furthermore, by presenting the experimental solution together with the control solution in a pairwise manner, we standardized the number of ants on an experimental solution with the number of ants on a control solution in statistical analyses (see below) to further control the possible variation in ants' activity among trails and nests.

After presentation, we counted the number of ants drinking from the different solutions every minute for the next 5 min (Reudler et al. 2015; Lindstedt et al. 2017), and started this observation period when the first ant worker encountered either of the two droplets. Each ant nest (*N* = 9) was tested three times (*N* = 3) with pooled fluid samples collected from each sex (*N* = 2) within each maternal care treatment (*N* = 4). (*N* = 9 × 3 × 2 × 4 = 216 possible trials of which 213 yielded data). All tests were run within a 1 wk period in August 2011 in central Finland (62 °N, 26° E) in sunny and warm weather (15 to 20 °C).

*Nicrophorus vespilloides* adults have two stripes of conspicuous orange markings on their elytra (Figure S1), which we have previously suggested acts as an aposematic signal (Lindstedt et al. 2017). To measure the pattern size and color, we photographed frozen beetles using a calibrated Fuji IS digital camera, which records both ultraviolet and human visible signals. From the photographs, the size of the elytra and of the orange stripes were measured with ImageJ, following (Lindstedt et al. 2017). In addition, we measured the brightness and saturation of their orange and black color to potential bird predators transformed to cone catch data of blue tits (*Cyanistes caeruleus*). To do that, we chose approximately similar sized square shaped samples (region of interests, ROIs) from the left side of the beetle's elytra from each of the orange stripes as well as from the black markings. The hue and brightness of the orange and black markings were analyzed with the Image Calibration and Analysis Toolbox (Koski et al. 2017), using the method described in (Lindstedt et al. 2017). We calculated saturation values (color richness), and brightness (double cone sensitivity) after (María Arenas et al. 2015) for the ROIs of the first and second orange stripes and black pattern.

### Statistics

All phenotypic traits ie body size, the size and color (saturation and brightness) of the orange signal and the quantity and quality of the defensive fluid, were analyzed using linear mixed effects models using the *lme4* and *lmerTest* packages (Bates et al. 2014; Kuznetsova et al. 2017) in the *R* statistical package version 3.2.2 (R Core Team 2013). Initial data exploration suggested that most traits of interest increased linearly with the duration of care. Therefore, treatment was coded as a continuous variable corresponding to the *Duration of care* in hours for all models reported below. In all cases we fitted the maximal model with all interaction terms included and used stepwise deletion based on their Type 3 sums of squares until we were left with significant terms only. From the final models we routinely report terms for Type 3 sums of square except otherwise noted. The maximal models with final and deleted terms are reported in supplementary Tables S1 to S3. The residuals from each model were visually examined for deviations from normality and for heteroscedasticity (Zuur et al. 2010).

### Body size, warning signal size, warning signal salience and volume of the defensive fluid

For every model, the *family* from which the beetles were derived, nested within *treatment*, was included as a random effect. To analyze how maternal care affected the size of the beetles, we considered the effects of *Duration of care* and *Sex* on the variation in body mass and elytra size. For the color traits and defense fluid quantity, we additionally considered the effects of *Elytra size* although the results were very similar if we included *Body mass* in its place. The saturation of the orange stripes showed significant deviations from normality and so were transformed using the transform Tukey function in the *Rcompanion* package (Mangiafico 2017), which uses Tukey's ladder of powers to iteratively select the best transformation (selected transformation = *x*^8.05^, *P* < 0.001).

### Repellence of the defensive fluid

To test the effect of *Duration of care in hours* and *Sex* of the beetle on the repellence of the defense fluid against ants we used a generalized linear mixed effects model with a binomial family. The response variable was a matrix of ants drinking sugar vs ants drinking exudate created using the cbind function in R. Treatment was again coded as a continuous variable with *trial number* and *sex* of the beetle from which the exudate originated included as factors. *Ant nest* was included as a random factor. As exudates were pooled within families, we did not need to account for the random effect of family in this analysis.

### Correlation between warning signal and volume of defensive fluid

To estimate the correlation between signal size, color and volume of defensive fluid, we used the MCMCglmm package in R to fit bivariate linear mixed models in a MCMC framework. In each case the bivariate response variable was a combination of *Defense fluid volume* and one of (1) *size of the orange pattern*, (2) *Saturation* of the orange pattern, and (3) *Brightness* of the orange pattern (luminance, ie, double cone sensitivity). For each bivariate combination of responses, we fitted four separate models, in each case using subsets of families receiving different levels of parental care. In each model, we fitted *Sex* and *Body size* (mg) as fixed covariates, plus a family-level random effect to estimate the family correlation. For the size of the orange pattern (OT) and defense fluid volume, we ran models including and excluding the outlier family (see Fig. 3d). We report the results without the outlier family even though including or excluding it does not change the results (see Figure S3 in Supplementary).

We fitted each model using parameter-expanded priors for the residual variance structure with nu = 2 (T. Houslay, pers. comm.). We also ran the models under a range of alternative priors for the residual component of the model (eg informative priors assuming equally apportioned variance, inverse-Wishart distributed priors) to check for robustness to starting assumptions. Three independent chains were run for each model and these were all checked by eye for autocorrelation and convergence with no issues.

## Results

### Body size, volume, and repellence of defensive fluid

**Fig. 1.**
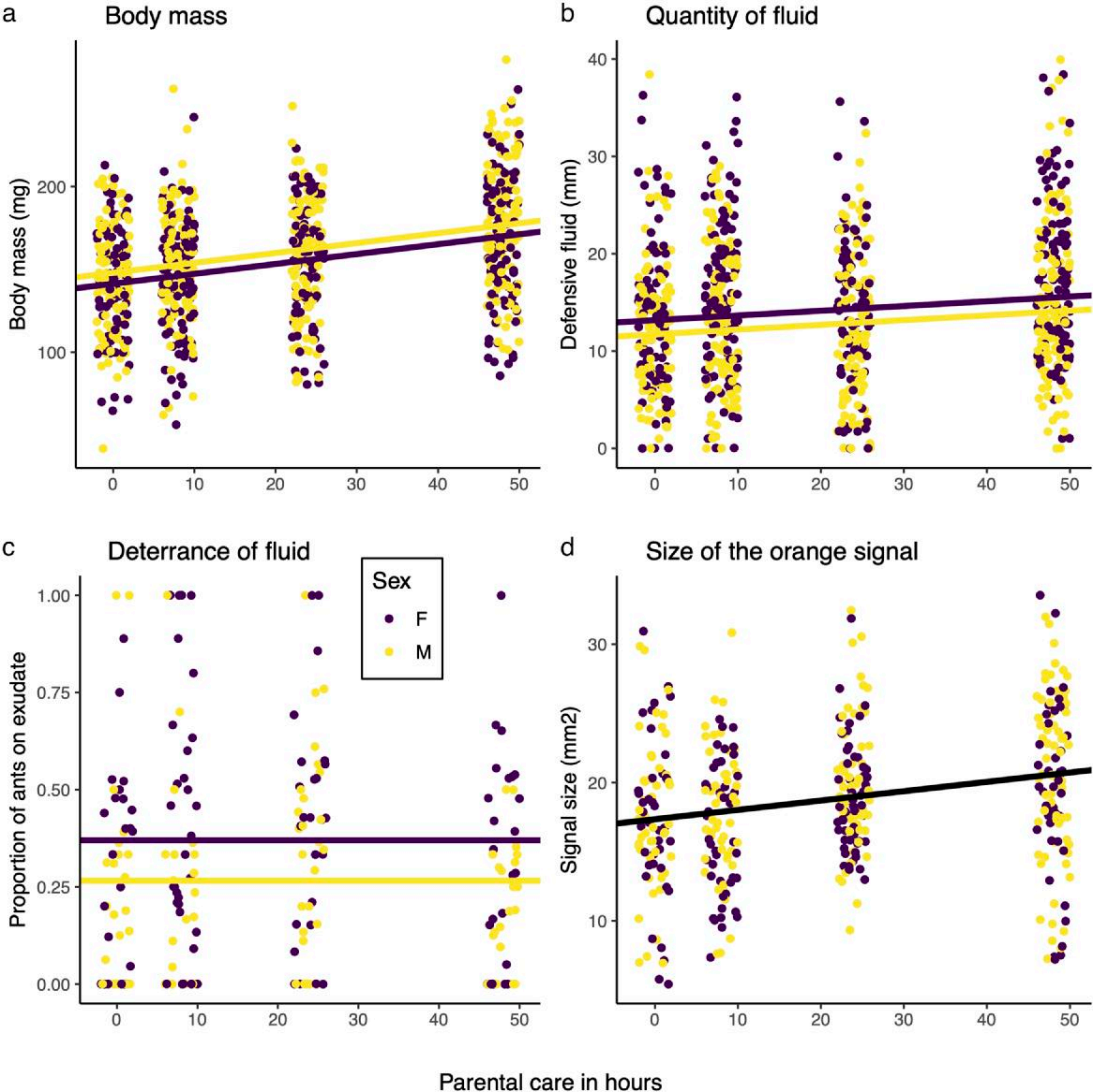
The effect of the duration of care on a) adult body mass, b) the quantity of the defensive fluid, c) the quality of the defensive fluid measured via its repellence to ants, and d) the total area of the orange signal for males and females. In each case, the fitted lines are predictions from the respective GLMM, results of which can be found in Tables S1 and S2.

In general, we found that maternal care affected the quality of the offspring. The greater the duration of care, the greater the adult body mass attained by offspring of both sexes (Table S1; Fig. 1a), and the larger the elytra (Table S1; Figure S2a). Elytra size did not differ between the sexes (sex: *F*_1,374_ = 0.27, *P* = 0.61; Figure S2a), but males were significantly heavier than females (Table S1; Fig. 1a). The interaction between care duration and sex was not significant for either trait (Table S1).

The volume of defensive fluid exuded under threat increased with the duration of care (Table S2; Fig. 1b), but this effect was mainly driven by the effect of maternal care on beetle size (Elytra size: *F*_1,324_ = 21.38, *P* < 0.001). After accounting for elytra size in the model, this effect disappeared (Table S2). Females produced more fluid than males and this was independent of size (Table S2, Fig. 1b). The amount of maternal care did not significantly affect the repellence of the defensive fluid, but ants found males exudate more aversive than that produced by females (Table S2, Fig. 1c).

### Warning signal size

As with the volume of defensive fluid, we found that the absolute size of the orange warning signal pattern increased with the level of care provided (Table S2; Fig. 1d). However, after accounting for elytra size in the model, this effect disappeared (Table S2), suggesting that the effect was driven by the increase in beetle size caused by greater levels of care (Fig. 1a, Figure S2a). The size of the orange signal was larger in males even after controlling statistically for elytra size (Table S2; Fig. 1d).

To test whether beetles of all sizes produced a proportionally similar signal, we looked at whether the relative signal size (area of the orange signal divided by the length of the elytron) changed with elytron length. We found that as beetles got larger, the relative signal size increased (Elytra size: *F*_1,410_ = 20.26, *P* < 0.001; Figure S2b) suggesting that larger beetles invested relatively more into the size of the orange signal than small beetles.

### Warning signal salience

**Fig. 2.**
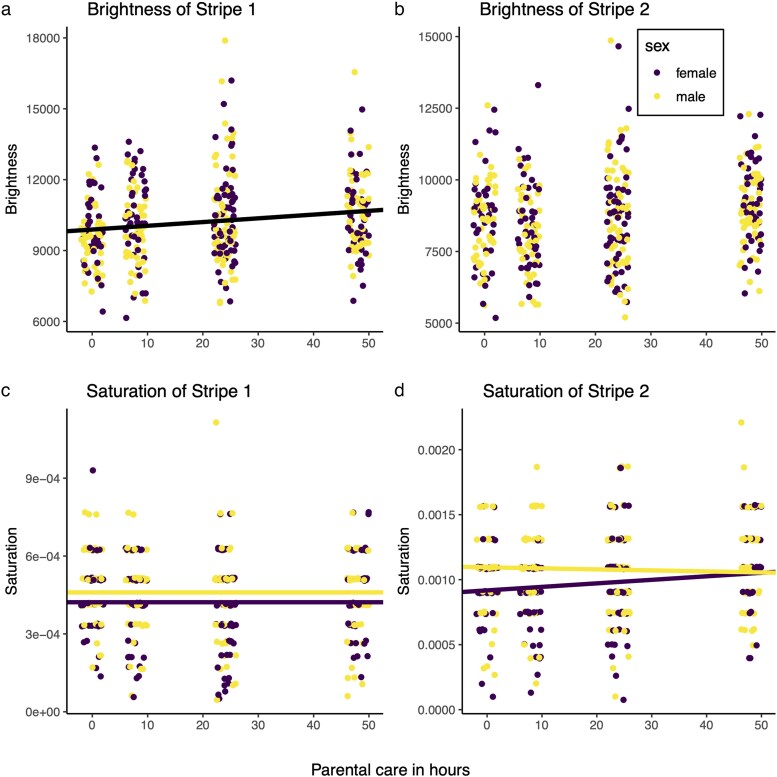
The effect of the duration of care on signal salience measured as brightness a, b) and saturation c, d) of the orange signal, measured separately for stripe 1 a, c) and stripe 2 b, d). Females are in purple and males in yellow. In each case, the fitted lines are predictions from the respective GLMM, results of which can be found in Table S3.

The brightness of the first orange stripe increased with the duration of care received as a larva, independently of the beetle's size and sex (Table S3; Fig. 2a). In contrast, the brightness of the smaller, second orange stripe was not affected by the duration of care or the beetle's sex (Table S3; Fig. 2b), but it did increase with the size of the beetle (Table S3).

The saturation of the first orange stripe was not affected by the duration of care received as a larva, but it did increase with beetle size and was slightly higher in males (Table S3; Fig. 2c). The saturation of the second orange stripe also increased with beetle size and, for females, also increased with the duration of care. In contrast, for males, saturation of the second stripe decreased slightly with the duration of care beetles experienced. Males had more saturated second stripes than females (Table S3, Fig. 2d).

The brightness and saturation of the black pattern on the elytra were not explained by sex, elytra size or duration of care or the interactions between any of the variables (Table S3).

### Correlations between the warning coloration and chemical defense

We analyzed the correlation between the visual signal (size and color) and the volume of defensive fluid to test whether the amount of maternal care received affected the information content of offspring's warning coloration. We fitted separate bivariate MCMC models within sets of broods receiving different parental care treatments to estimate the correlation at the brood level between the amount of defensive fluid produced and the intensity of the various signal components. Then we compared the brood-level correlation coefficient among these models.

**Fig. 3.**
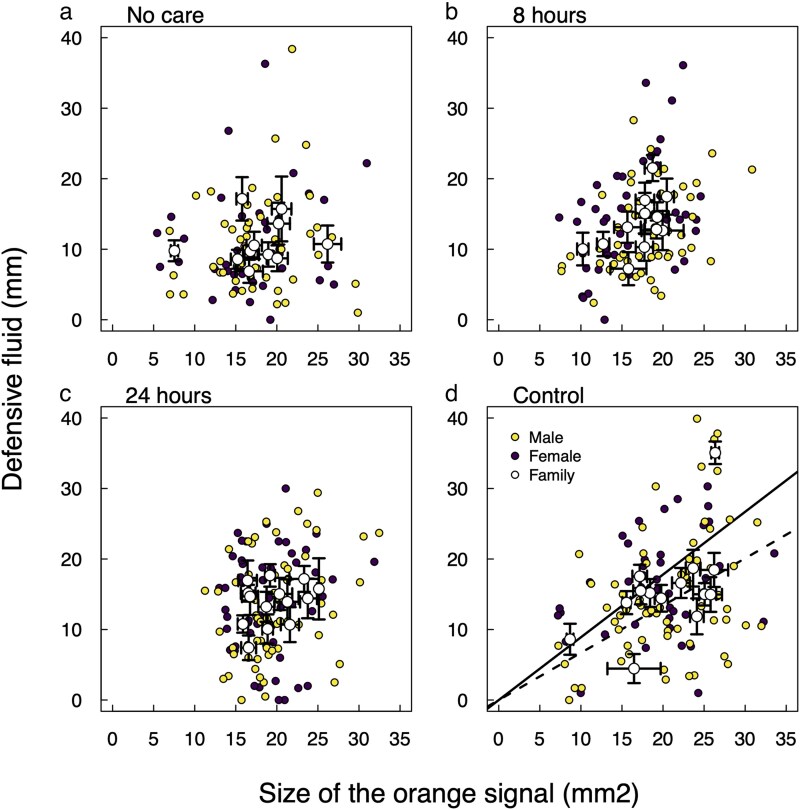
A representation of the family level correlation between signal size and the quantity of the defensive fluid produced for a) No care, b) 8 h of care, c) 24 h of care, and d) 48 h (control) of care beetles. Individual data points for males and females are in yellow and purple respectively with the family level average ± SE included in white. The family correlation (black line) was estimated using MCMCglmm and was significant for the 48 h of care treatment only. The dotted line represents the family level correlation when the outlier family in the top right was included, and solid line with it removed.

According to these models, the correlation between defensive fluid volume and brightness was never significantly different from zero (Table 1). Correlations between defensive fluid volume and both (1) the total area of orange patterning and (2) the saturation of this orange coloration were also indistinguishable from zero in all broods—except for broods that received the maximum level of care (Table 1, Fig. 3 and Figure S3). In these broods, the correlation with the volume of exudate produced was positive for both measures of the visual display. This means, that under full parental care, the broods that on average displayed larger patches of orange, which were more saturated in color, also produced higher quantities of defensive fluid. The level of care received as a larva thus modulates the reliability of the aposematic display in adulthood.

**Table 1.** Family-level and residual structure in bivariate MCMC models of (1) defensive fluid (DF) and total area of orange (OT), (2) DF and OT excluding one outlier family (see Fig. 3); and (3) DF and saturation of the first stripe [SAT], fitted using the MCMCglmm package in R.

	Family-level structure				Residual structure			
(a)	Family Var (DF)	Family Corr (DF, OT)	Family Cov (DF, OT)	Family Var (OT)	Residual Var (DF)	Residual Corr (DF, OT)	Residual Cov (DF, OT)	Residual Var (OT)
**0 h**	0.12 (4.9 × 10^−05^ to 16)	−0.27 (−0.88 to 0.64)	0.0047 (−10 to 9.1)	16 (6.7 to 55)	48 (32 to 60)	−0.27 (−0.88 to 0.64)	0.4 (−3 to 4.8)	6 (4.2 to 8)
**8 h**	0.083 (2.6 × 10^−05^ to 16)	0.11 (−0.53 to 0.88)	0.45 (−4.5 to 7.7)	7.6 (2.9 to 26)	35 (26 to 49)	0.11 (−0.53 to 0.88)	3 (−0.79 to 6.4)	7.7 (5.4 to 10)
**24 h**	0.22 (0.00036 to 20)	0.15 (−0.79 to 0.81)	−0.012 (−3.2 to 2.4)	0.52 (1.6 × 10^−05^ to 4.3)	47 (36 to 60)	0.15 (−0.79 to 0.81)	2.8 (−1.5 to 6.1)	8.6 (6 to 10)
**48 h**	31 (10 to 100)	0.67 (0.00058 to 0.91)	10 (−3.9 to 37)	16 (5.7 to 38)	41 (31 to 55)	0.67 (0.00058 to 0.91)	0.86 (−3.1 to 4)	6.7 (5 to 8.8)

(b)	Family Var (DF)	Family Corr (DF, OT)	Family Cov (DF, OT)	Family Var (OT)	Residual Var (DF)	Residual Corr (DF, OT)	Residual Cov (DF, OT)	Residual Var (OT)
**0 h**	0.098 (5e−08 to 17)	−0.12 (−0.87 to 0.67)	−0.064 (−12 to 7.4)	17 (5.8 to 55)	44 (33 to 61)	−0.12 (−0.87 to 0.67)	0.47 (−2.9 to 5)	6.2 (4.2 to 8.2)
**8 h**	0.17 (3.4e−06 to 17)	0.35 (−0.58 to 0.86)	0.048 (−5.7 to 8.5)	7.9 (3 to 26)	35 (27 to 49)	0.35 (−0.58 to 0.86)	2.4 (−0.84 to 6.8)	7.4 (5.6 to 10)
**24 h**	0.051 (2.2e−05 to 21)	−0.16 (−0.78 to 0.76)	0.0012 (−2.9 to 2.7)	0.71 (3.2e−05 to 3.9)	45 (34 to 60)	−0.16 (−0.78 to 0.76)	2.1 (−1.6 to 6.1)	8 (5.9 to 10)
**48 h**	9.4 (0.93 to 30)	0.89 (0.24 to 1)	7.8 (0.16 to 25)	13 (6.4 to 39)	39 (30 to 54)	0.89 (0.24 to 1)	0.7 (−2.7 to 4.5)	6.8 (5.1 to 9.1)

(c)	Family Var (DF)	Family Corr (DF, SAT)	Family Cov (DF, SAT)	Family Var (SAT)	Residual Var (DF)	Residual Corr (DF, SAT)	Residual Cov (DF, SAT)	Residual Var (SAT)
**0 h**	0.15 (3.6 × 10^−06^ to 17)	0.38 (−0.77 to 0.89)	−6.5 × 10^−05^ (−0.0075 to 0.012)	−9.5 × 10^−07^ (4.2 × 10^−12^ to 6.9 × 10^−05^)	40 (33 to 61)	0.38 (−0.77 to 0.89)	0.0062 (−0.03 to 0.025)	0.00029 (0.00021 to 0.00039)
**8 h**	0.09 (0.00014 to 17)	−0.081 (−0.95 to 0.77)	6.2 × 10^−06^ (−0.012 to 0.0096)	5.3 × 10^−06^ (1.3 × 10^−11^ to 8 × 10^−05^)	39 (26 to 49)	−0.081 (−0.95 to 0.77)	0.0086 (−0.028 to 0.034)	0.00056 (0.00037 to 7 × 10^−04^)
**24 h**	2.4 (0.00021 to 19)	−0.75 (−0.99 to 0.54)	4.1 × 10^−05^ (−0.035 to 0.014)	1.5 × 10^−06^ (1.3 × 10^−09^ to 0.00034)	47 (33 to 60)	−0.75 (−0.99 to 0.54)	0.0066 (−0.042 to 0.053)	0.0011 (0.00082 to 0.0014)
**48 h**	33 (13 to 90)	0.92 (0.048 to 1)	0.032 (−0.011 to 0.15)	8.2 × 10^−05^ (1.1 × 10^−07^ to 0.00041)	41 (29 to 53)	0.92 (0.048 to 1)	−0.012 (−0.035 to 0.019)	0.00039 (0.00027 to 0.00051)

Values are the posterior mode of each distribution with the upper and lower confidence 95% intervals in parentheses. In each case models were fitted separately for each experimental treatment, and in addition to family as a random effect, body size and sex were fitted as fixed effects (data not shown).

## Discussion

Our results show that the level of care provided by mothers can have long-term effects on the development and information content of aposematic displays. First, we found that the greater the level of care received by larvae, the more salient was the visual warning signal to predators in adulthood (brightness in both sexes and color saturation in females). Furthermore, individuals that received more care matured into larger adults, displayed correspondingly larger warning signal patterns, and produced greater volumes of defensive fluid when subjected to simulated attack. Therefore, a key new insight from our study is that parental effects may offer protection against predators by enhancing the salience and potency of an aposematic display borne in adulthood either directly (brightness and saturation of color) and/or indirectly due to a larger size.

Second, we found that the size of an individual's visual warning display was positively correlated among broods with the extent of the chemical defense, but only in families that received full maternal care as larvae. For families receiving full care, our results contribute to the increasing evidence that warning signal expression correlates positively with the extent of chemical defenses ie that warning signals can be quantitatively reliable (White and Umbers 2021). At the same time, our results show that when there is a reduced supply of care, pattern size and its saturation may become a less reliable signal of chemical defenses. Maternal care thus modulates the reliability of the aposematic display and, in so doing, modulates exposure of aposematic signal reliability to selection by predators: the lower the level of care supplied, the less likely it is that a reliable warning display is exposed to selection.

We can think of two main explanations for a decay in the correlation between the visual display and the strength of the chemical defenses at low levels of care. These mechanisms are not mutually exclusive and likely to operate simultaneously, by increasing within-family variation (Table 1). First, low levels of maternal care are more stressful for offspring and increase environmental variation in their chemical defenses (residual variation for defensive fluid quantity is higher and family-level variation lower in comparison to color variables in reduced care treatments, Table 1.), thus reducing warning signal reliability (Ebert et al. 1993; Hoffmann and Merilä 1999). For example, within reduced-care families (where larvae are in broadly poorer condition), some larvae may have monopolized the resource (or were just better at self-feeding). These individuals ended up with enough resources to become large and to bear a large orange elytral pattern and were also able to invest in high volumes of defensive fluid. Some of their siblings may still have had relatively large orange signals but due to stressful conditions (increased competition for food/certain nutrients or poor ability for self-feeding) could not invest in high volumes of defensive fluid. Many important pigment groups such as carotenoids, melanins and pteridines can potentially function as antioxidants (Blount et al. 2009, 2012) and they are therefore needed also for the protective functions for autotoxicity (Blount et al. 2009), immune responses (Ojala et al. 2005) and oxidative stress during growth (Flores et al. 2013). Even though we do not have information about the metabolic processes underlying the pigmentation in *N. vespilloides* burying beetles, perhaps under more stressful reduced parental care conditions, antioxidant defenses are more quickly depleted, resulting in the decay of signal reliability. This hypothesis is also supported by studies from other *Nicrophoru*s species (*N. pustulatus*) which show that individuals with more red pigmentation in their clypeal membrane color signals have lower immune responses suggesting that the production of red pigmentation is physiologically costly (Wormington and Luttbeg 2018, 2019).

Secondly, the pattern of covariation between signal salience and chemical defense could be driven by parental effects. For example, parents could selectively provide extra nutrients to smaller larvae (Smiseth et al. 2007), thus reducing within-brood variation under full care in comparison to those receiving reduced care or no care at all. Because parents vary in quality, the absolute amount of resources each larva within a brood receives is likely to vary with the quality of the parent, resulting in family-level variation in aposematic traits under full care (Fig. 1). This explanation is consistent with previous work with seven spot ladybird beetle (*Coccinella septempunctata*) suggesting that maternal investment of pigments into the eggs correlates positively with the hue and saturation of newly eclosed adults and may influence on the honesty of adult warning coloration (Winters et al. 2014). Similarly, the early-life environment experienced by tiger moths influences the potency of their chemical defenses, since they are weaker when larvae were food deprived (Burdfield-Steel et al. 2019).

More generally, our results suggest that we can expect quantitatively reliable warning signals to be more likely to evolve under well-resourced developmental environments. This is partly in conflict with predictions from some of the previous theoretical models (Leimar et al. 1986; Blount et al. 2009) which have considered the effect of resource limitation on warning signal honesty. These models predict a negative correlation between the conspicuousness of the warning signal and extent of chemical defense under unlimited resource environment, and a positive correlation under limited resources. In contrast, we found that signals were honest on average in the high resource environment (unlimited parental care), but the positive association degraded under reduced resources as individuals ended up investing asymmetrically in defensive traits. Notably, the deterrence of defensive exudate was not affected by the resources provided via parental care. One potential explanation for this finding is that under limited resources organisms allocate resources in a hierarchical manner that is often genetically determined (de Jong 1993; Nijhout and Emlen 1998; Simmons and Emlen 2006). Traits that play a more critical role in terms of individual fitness appear to be prioritized for investment when nutritional conditions are poor (de Jong 1993; Nijhout and Emlen 1998). For example, studies with the aposematic wood tiger moths have shown that under limited resources investment in highly heritable warning signal pattern does not change, but individuals invest less on the extent of chemical defense (Burdfield-Steel et al. 2019) or life-history traits (Lindstedt et al. 2020b). It is possible that in *N. vespilloides,* the association between signal salience and quantity of defensive exudate breaks down under reduced parental care as individuals prioritize resources among traits differently than in the full parental care treatment. However, we note that it is difficult to determine the position of our control treatment on a resource limitation continuum as even when maternal care was unlimited, the availability of nutrients may have been affected by other factors such as the quality of the carrion nest.

Interestingly, we found sexual dimorphism both in the salience of the visual display (males were more saturated in orange coloring than females) and in the chemical defenses (females produced more fluid in response to attack but defensive fluids of males were more noxious to ants). These sex differences might simply reflect different sex-specific roles in reproduction and parental care (Lindstedt et al. 2017). Previous work has found that females allocate more effort to the maintenance of the carcass during biparental care, which might involve producing higher quantities of anal exudate to spread on the carcass. By contrast, males put more effort defending the carcass against other beetles and other competitors such as ants and flies (Rauter and Moore 2004; Smiseth and Moore 2004; Trumbo 2007; Fetherston et al. 2018). This might explain why the exudates males produce are more repellent. We also note that sexual dimorphism in saturation of the orange pattern indicates that orange-black coloration may function in intraspecific signaling of individual's condition (see also Wormington and Luttbeg 2019) in addition to its potential warning signal function providing an interesting avenue for future research.

Our results also open up further questions for future research. First, it is unknown whether the quantity or the quality of the responsive chemical defense is the more important for predators in determining the information content and reliability of the warning signals. Our bioassays with ants indicated that maternal care did not affect the deterrence of chemical defenses in adult life (ie quality of the defensive exudate). However, it is important to note that the exudate that *N. vespilloides* produce under threat is highly deterrent even in low concentrations (Lindstedt et al. 2017). As a result, being able to produce a greater volume of this highly potent defense might offer the more profitable route to further strengthening antipredator defenses. Secondly, hardly any studies have investigated how the average reliability of the warning signal influences long-term survival under a natural predator community structure (White and Umbers 2021). *N. vespilloides* potentially offers a good model system for investigating this problem since it is feasible to produce individuals from different parental care treatments and conduct mark-recapture experiments to measure their survival in the wild.

In conclusion, our results add to the accumulating experimental evidence that extended parental care plays a central role in buffering offspring against different type of environmental hazards, both in *Nicrophorus* spp. (Rozen et al. 2008; Mattey et al. 2018; Grew et al. 2019; Malik et al. 2025) and other species (Clutton-Brock 1991; Royle et al. 2012). Furthermore, previous studies have shown that the social environment can impose selection on traits that mediate social interactions *within species* (West-Eberhard 1983; Lande and Kirkpatrick 1990; Tanaka 1996; Wolf et al. 1999). Here we provide evidence that the social environment can also influence communication *between species* by modulating warning signal reliability.

Together, these results suggest that different modes of parental care can contribute to an organism's ability to persist in a rapidly changing ecological and abiotic conditions or expand their distribution range to more extreme or harsher conditions (Cornwallis et al. 2017; Mattey et al. 2018; Grew et al. 2019; Komdeur and Ma 2021; Boucicot et al. 2025; Malik et al. 2025). More generally, our results further suggest that to explain intraspecific diversity in adaptive traits such as warning displays, we need to consider multiple agents whose behavior affects selection on the trait of interest (Lindstedt et al. 2020b) (in this case such agents are predators and mothers). This will help us move toward a more realistic view of the complexity of the selective environments in which adaptive traits evolve and persist in nature.

## Supplementary Material

araf116_Supplementary_Data

## Data Availability

Analyses reported in this article can be reproduced using the data provided by (Lindstedt et al. 2025).

## References

[araf116-B1] Bates D, Mächler M, Bolker BM, Walker SC. 2014. Fitting linear mixed-effects models using lme4. J Stat Softw. 67:1–48. 10.18637/jss.v067.i01.

[araf116-B2] Beatty CD, Beirinckx K, Sherratt TN. 2004. The evolution of mullerian mimicry in multispecies communities. Nature. 431:63–66. 10.1038/nature02818. 15343332

[araf116-B3] Bleakley BH, Brodie ED 3rd. 2009. Indirect genetic effects influence antipredator behavior in guppies: estimates of the coefficient of interaction psi and the inheritance of reciprocity. Evolution. 63:1796–1806. 10.1111/j.1558-5646.2009.00672.x. 19245394

[araf116-B4] Blount JD et al 2012. How the ladybird got its spots: effects of resource limitation on the honesty of aposematic signals. Funct Ecol. 26:334–342. 10.1111/j.1365-2435.2012.01961.x.

[araf116-B5] Blount JD, Speed MP, Ruxton GD, Stephens PA. 2009. Warning displays may function as honest signals of toxicity. Proc Biol Sci. 276:871–877. 10.1098/rspb.2008.1407. 19019790 PMC2664363

[araf116-B6] Boncoraglio G, Kilner RM. 2012. Female burying beetles benefit from male desertion: sexual conflict and counter-adaptation over parental investment. PLoS One. 7:e31713. 10.1371/journal.pone.0031713. 22355390 PMC3280230

[araf116-B7] Boucicot M et al 2025. Earwig mothers can boost offspring's defence against pathogens during postoviposition care. Anim Behav. 219:123010. 10.1016/j.anbehav.2024.10.024.

[araf116-B8] Briolat ES et al 2019. Diversity in warning coloration: selective paradox or the norm? Biol Rev Camb Philos Soc. 94:388–414. 10.1111/brv.12460. 30152037 PMC6446817

[araf116-B9] Brown JL, Morales V, Summers K. 2010. A key ecological trait drove the evolution of biparental care and monogamy in an amphibian. Am Nat. 175:436–446. 10.1086/650727. 20180700

[araf116-B10] Burdfield-Steel E, Brain M, Rojas B, Mappes J. 2019. The price of safety: food deprivation in early life influences the efficacy of chemical defence in an aposematic moth. Oikos. 128, 245–253. 10.1111/oik.05802.

[araf116-B11] Chatelain P et al 2023. Müllerian mimicry among bees and wasps: a review of current knowledge and future avenues of research. Biol Rev Camb Philos Soc. 98:1310–1328. 10.1111/brv.12955. 36994698

[araf116-B12] Clutton-Brock TH . 1991. The evolution of parental care. Princeton University Press. 368 p.

[araf116-B13] Conner W . 2009. Tiger moths and woolly bears: behavior, ecology, and evolution of the arctiidae. Oxford University Press. 303 p.

[araf116-B14] Cornwallis CK et al 2017. Cooperation facilitates the colonization of harsh environments. Nat Ecol Evol. 1:0057. 10.1038/s41559-016-0057. 28812731

[araf116-B15] Cotter SC, Kilner RM. 2010. Personal immunity versus social immunity. Behav Ecol. 21:663–668. 10.1093/beheco/arq070.

[araf116-B16] De Gasperin O, Duarte A, Troscianko J, Kilner RM. 2016. Fitness costs associated with building and maintaining the burying beetle's carrion nest. Sci Rep. 6:35293. 10.1038/srep35293. 27734965 PMC5062497

[araf116-B17] Degenkolb T, Düring R-A, Vilcinskas A. 2011. Secondary metabolites released by the burying beetle Nicrophorus vespilloides: chemical analyses and possible ecological functions. J Chem Ecol. 37:724–735. 10.1007/s10886-011-9978-4. 21667150

[araf116-B18] De Jong G . 1993. Covariances between traits deriving from successive allocations of a resource. Funct Ecol. 7:75–83. 10.2307/2389869.

[araf116-B19] Ebert D, Yampolsky L, Stearns SC. 1993. Genetics of life history in Daphnia magna. I. Heritabilities at two food levels. Heredity (Edinb). 70:335–343. 10.1038/hdy.1993.48.

[araf116-B20] Eggert A-K, Reinking M, Müller JK. 1998. Parental care improves offspring survival and growth in burying beetles. Anim Behav. 55:97–107. 10.1006/anbe.1997.0588. 9480676

[araf116-B21] Fetherston IA, Scott MP, Traniello JFA. 2018. Parental care in burying beetles: the organization of male and female brood-care behavior. Ethology. 85:177–190. 10.1111/j.1439-0310.1990.tb00398.x.

[araf116-B22] Flores EE, Stevens M, Moore AJ, Blount JD. 2013. Diet, development and the optimization of warning signals in post-metamorphic green and black poison frogs. Funct Ecol. 27:816–829. 10.1111/1365-2435.12084.

[araf116-B23] Giesing ER, Suski CD, Warner RE, Bell AM. 2011. Female sticklebacks transfer information via eggs: effects of maternal experience with predators on offspring. Proc Biol Sci. 278:1753–1759. 10.1098/rspb.2010.1819. 21068041 PMC3081764

[araf116-B24] Grew R, Ratz T, Richardson J, Smiseth PT. 2019. Parental care buffers against effects of ambient temperature on offspring performance in an insect. Behav Ecol. 30:1443–1450. 10.1093/beheco/arz100.

[araf116-B25] Higginson AD, Delf J, Ruxton GD, Speed MP. 2011. Growth and reproductive costs of larval defence in the aposematic lepidopteran Pieris brassicae. J Anim Ecol. 80:384–392. 10.1111/j.1365-2656.2010.01786.x. 21155771

[araf116-B26] Hoffmann AA, Merilä J. 1999. Heritable variation and evolution under favourable and unfavourable conditions. Trends Ecol Evol. 14:96–101. 10.1016/S0169-5347(99)01595-5. 10322508

[araf116-B27] Holen ØH, Svennungsen TO. 2012. Aposematism and the handicap principle. Am Nat. 180:629–641. 10.1086/667890. 23070323

[araf116-B28] Jarrett BJM et al 2018. A sustained change in the supply of parental care causes adaptive evolution of offspring morphology. Nat Commun. 9:3987. 10.1038/s41467-018-06513-6. 30266903 PMC6162320

[araf116-B29] Jarrett BJM, Schrader M, Rebar D, Houslay TM, Kilner RM. 2017. Cooperative interactions within the family enhance the capacity for evolutionary change in body size. Nat Ecol Evol. 1:0178. 10.1038/s41559-017-0178. 28685165 PMC5495167

[araf116-B30] Kapan DD . 2001. Three-butterfly system provides a field test of müllerian mimicry. Nature. 409:18–20. 10.1038/35053066. 11201741

[araf116-B31] Kilner RM et al 2015. Parental effects alter the adaptive value of an adult behavioural trait. Elife. 4:e07340. 10.7554/eLife.07340. 26393686 PMC4613925

[araf116-B32] Komdeur J, Ma L. 2021. Keeping up with environmental change: the importance of sociality. Ethology. 127:790–807. 10.1111/eth.13200.

[araf116-B33] Koski TM et al 2017. Insect herbivory may cause changes in the visual properties of leaves and affect the camouflage of herbivores to avian predators. Behav Ecol Sociobiol. 71:97. 10.1007/s00265-017-2326-0.

[araf116-B34] Kozak GM, Boughman JW. 2012. Plastic responses to parents and predators lead to divergent shoaling behaviour in sticklebacks. J Evol Biol. 25:759–769. 10.1111/j.1420-9101.2012.02471.x. 22320242

[araf116-B35] Kuznetsova A, Brockhoff PB, Christensen RHB. 2017. lmerTest package: tests in linear mixed effects models. J Stat Softw. 82:1–126. 10.18637/jss.v082.i13.

[araf116-B36] Lande R, Kirkpatrick M. 1990. Selection response in traits with maternal inheritance. Genet Res. 55:189–197. 10.1017/S0016672300025520. 2394377

[araf116-B37] Lane C, Rothschild M. 1965. A case of müllerian mimicry of sound. Proc R Entomol Soc Lond Ser A Gen Entomol. 40:156–158. 10.1111/j.1365-3032.1965.tb00305.x.

[araf116-B38] Leimar O, Enquist M, Sillen-Tullberg B. 1986. Evolutionary stability of aposematic colouration and prey unprofitability—a theoretical analysis. Am Nat. 128:469–490. 10.1086/284581.

[araf116-B39] Lindstedt C et al 2011a. Direction and strength of selection by predators for the color of the aposematic wood tiger moth. Behav Ecol. 22:580–587. 10.1093/beheco/arr017.

[araf116-B40] Lindstedt C et al 2018. Ecological conditions alter cooperative behaviour and its costs in a chemically defended sawfly. Proc Biol Sci. 285:20180466. 10.1098/rspb.2018.0466. 30068673 PMC6111175

[araf116-B41] Lindstedt C, Bagley RK, Calhim S, Jones M, Linnen CR. 2022. The impact of life stage and pigment source on the evolution of novel warning signal traits. Evolution. 76:554–572. 10.1111/evo.14443. 35103303 PMC9304160

[araf116-B42] Lindstedt C, Boncoraglio G, Cotter S, Gilbert J, Kilner RM. 2017. Aposematism in the burying beetle? Dual function of anal fluid in parental care and chemical defence. Behav Ecol. 28:1414–1422. 10.1093/beheco/arx100.

[araf116-B43] Lindstedt C, Boncoraglio G, Cotter S, Gilbert J, Kilner RM. 2025. Data from: maternal care shapes an aposematic display and provides lifelong protection against predators. Behav Ecol. 10.5061/dryad.1jwstqk80. PMC12541378 41132817

[araf116-B44] Lindstedt C, Huttunen H, Kakko M, Mappes J. 2011b. Disengtangling the evolution of weak warning signals: high detection risk and low production costs of chemical defences in gregarious pine sawfly larvae. Evol Ecol. 25:1029–1046. 10.1007/s10682-010-9456-4.

[araf116-B45] Lindstedt C, Lindström L, Mappes J. 2008. Hairiness and warning colours as components of antipredator defence: additive or interactive benefits? Anim Behav. 75:1703–1713. 10.1016/j.anbehav.2007.10.024.

[araf116-B46] Lindstedt C, Mappes J, Päivinen J, Varama M. 2006. Effects of group size and pine defence chemicals on Diprionid sawfly survival against ant predation. Oecologia. 150:519–526. 10.1007/s00442-006-0572-3. 16924548

[araf116-B47] Lindstedt C, Suisto K, Burdfield-Steel E, Winters AE, Mappes J. 2020a. Defense against predators incurs high reproductive costs for the aposematic moth Arctia plantaginis. Behav Ecol. 31:844–850. 10.1093/beheco/araa033. 32595271 PMC7303824

[araf116-B48] Lindstedt C, Suisto K, Mappes J. 2020b. Appearance before performance? Nutritional constraints on life-history traits, but not warning signal expression in aposematic moths. J Anim Ecol. 89:494–505. 10.1111/1365-2656.13103. 31538333 PMC7027542

[araf116-B49] Lock JE, Smiseth PT, Moore AJ. 2004. Selection, inheritance, and the evolution of parent-offspring interactions. Am Nat. 164:13–24. 10.1086/421444. 15266367

[araf116-B50] Maan ME, Cummings ME. 2009. Sexual dimorphism and directional sexual selection on aposematic signals in a poison frog. Proc Natl Acad Sci U S A. 106:19072–19077. 10.1073/pnas.0903327106. 19858491 PMC2776464

[araf116-B51] Malik TG, Tsai M-T, Jarrett BJM, Sun S-J. 2025. Heat stress effects on offspring compound across parental care. Proc Biol Sci. 292:20250026. 10.1098/rspb.2025.0026. 40041959 PMC11881022

[araf116-B52] Mallet J, Barton NH. 1989. Strong natural selection in a warning-color hybrid zone. Evolution. 43:421–431. 10.1111/j.1558-5646.1989.tb04237.x. 28568556

[araf116-B53] Mangiafico S . rcompanion: Functions to Support Extension Education Program Evaluation in R, version 1.23.0, revised 2025. 2017.

[araf116-B54] Mappes J, Kokko H, Ojala K, Lindström L. 2014. Seasonal changes in predator community switch the direction of selection for prey defences. Nat Commun. 5:5016. 10.1038/ncomms6016. 25247589 PMC4199109

[araf116-B55] María Arenas L, Walter D, Stevens M. 2015. Signal honesty and predation risk among a closely related group of aposematic species. Sci Rep. 5:11021. 10.1038/srep11021. 26046332 PMC4457162

[araf116-B56] Mattey SN, Richardson J, Ratz T, Smiseth PT. 2018. Effects of offspring and parental inbreeding on parent-offspring communication. Am Nat. 191:716–725. 10.1086/697236. 29750564

[araf116-B57] Moore AJ, Brodie ED 3rd, Wolf JB. 1997. Interacting phenotypes and the evolutionary process: I. Direct and indirect genetic effects of social interactions. Evolution. 51:1352–1362. 10.1111/j.1558-5646.1997.tb01458.x. 28568644

[araf116-B58] Naisbit RE, Jiggins CD, Mallet J. 2001. Disruptive sexual selection against hybrids contributes to speciation between Heliconius cydno and Heliconius melpomene. Proc Biol Sci. 268:1849–1854. 10.1098/rspb.2001.1753. 11522205 PMC1088818

[araf116-B59] Nijhout HF, Emlen DJ. 1998. Competition among body parts in the development and evolution of insect morphology. Proc Natl Acad Sci U S A. 95:3685–3689. 10.1073/pnas.95.7.3685. 9520426 PMC19896

[araf116-B60] Nishida R . 2002. Sequestration of defensive substances from plants by lepidoptera. Annu Rev Entomol. 47:57–92. 10.1146/annurev.ento.47.091201.145121. 11729069

[araf116-B61] Nokelainen O, Hegna RH, Reudler JH, Lindstedt C, Mappes J. 2012. Trade-off between warning signal efficacy and mating success in the wood tiger moth. Proc Biol Sci. 279:257–265. 10.1098/rspb.2011.0880. 21653589 PMC3223681

[araf116-B62] Ojala K, Julkunen-Tiitto R, Lindström L, Mappes J. 2005. Diet affects the immune defence and life-history traits of an Arctiid moth Parasemia plantaginis. Evol Ecol Res. 7:1153–1170.

[araf116-B63] Parker DJ et al 2015. Transcriptomes of parents identify parenting strategies and sexual conflict in a subsocial beetle. Nat Commun. 6:8449. 10.1038/ncomms9449. 26416581 PMC4598741

[araf116-B64] Parker GA, Royle NJ, Hartley IR. 2002. Intrafamilial conflict and parental investment: a synthesis. Philos Trans R Soc Lond B Biol Sci. 357:295–307. 10.1098/rstb.2001.0950. 11958698 PMC1692944

[araf116-B65] Pavelka LA, Kim YH, Mosher HS. 1977. Tetrodotoxin and tetrodotoxin-like compounds from the eggs of the Costa Rican frog, Atelopus Chiriquiensis. Toxicon. 15:135–139. 10.1016/0041-0101(77)90032-0. 854934

[araf116-B66] Pilakouta N, Hanlon EJH, Smiseth PT. 2018. Biparental care is more than the sum of its parts: experimental evidence for synergistic effects on offspring fitness. Proc Biol Sci. 285:20180875. 10.1098/rspb.2018.0875. 30068674 PMC6111165

[araf116-B67] Rauter CM, Moore AJ. 2004. Time constraints and trade-offs among parental care behaviours: effects of brood size, sex and loss of mate. Anim Behav. 68:695–702. 10.1016/j.anbehav.2003.09.018.

[araf116-B68] R Core Team . 2013. Statistical, R: a language and environment for computing. R Foundation for Statistical Computing.

[araf116-B69] Reudler JH, Lindstedt C, Pakkanen H, Lehtinen I, Mappes J. 2015. Costs and benefits of plant allelochemicals in herbivore diet in a multi enemy world. Oecologia. 179:1147–1158. 10.1007/s00442-015-3425-0. 26296333

[araf116-B70] Rojas B et al 2017. How to fight multiple enemies: target-specific chemical defences in an aposematic moth. Proc Biol Sci. 284:20171424. 10.1098/rspb.2017.1424. 28954910 PMC5627206

[araf116-B71] Royle N, Smiseth P, Kölliker M. 2012. The evolution of parental Care. Oxford University Press.

[araf116-B72] Rozen DE, Engelmoer DJP, Smiseth PT. 2008. Antimicrobial strategies in burying beetles breeding on carrion. Proc Natl Acad Sci U S A. 105:17890–17895. 10.1073/pnas.0805403105. 19001269 PMC2584725

[araf116-B73] Schrader M, Jarrett BJM, Kilner RM. 2015. Parental care masks a density-dependent shift from cooperation to competition among burying beetle larvae. Evolution. 69:1077–1084. 10.1111/evo.12615. 25648525 PMC4476075

[araf116-B74] Scott MP, Traniello JFA, Fetherston IA. 1987. Competition for prey between ants and burying beetles (Nicrophorus spp): differences between northern and southern temperate sites. Psyche (Stuttg). 94:325–332. 10.1155/1987/56594.

[araf116-B75] Sillén-Tullberg B . 1990. Do predators avoid groups of aposematic prey? An experimental test. Anim Behav. 40:856–860. 10.1016/S0003-3472(05)80986-8.

[araf116-B76] Simmons LW, Emlen DJ. 2006. Evolutionary trade-off between weapons and testes. Proc Natl Acad Sci U S A. 103:16346–16351. 10.1073/pnas.0603474103. 17053078 PMC1637585

[araf116-B77] Skelhorn J, Rowe C. 2006. Prey palatability influences predator learning and memory. Anim Behav. 71:1111–1118. 10.1016/j.anbehav.2005.08.011.

[araf116-B78] Smiseth PT, Moore AJ. 2004. Behavioral dynamics between caring males and females in a beetle with facultative biparental care. Behav Ecol. 15:621–628. 10.1093/beheco/arh053.

[araf116-B79] Smiseth PT, Ward RJS, Moore AJ. 2007. Parents influence asymmetric sibling competition: experimental evidence with partially dependent young. Ecology. 88:3174–3182. 10.1890/06-1992.1. 18229851

[araf116-B80] Speed MP, Ruxton GD, Blount JD, Stephens PA. 2010. Diversification of honest signals in a predator-prey system. Ecol Lett. 13:744–753. 10.1111/j.1461-0248.2010.01469.x. 20597158

[araf116-B81] Speed MP, Ruxton GD, Mappes J, Sherratt TN. 2012. Why are defensive toxins so variable? An evolutionary perspective. Biol Rev Camb Philos Soc. 87:874–884. 10.1111/j.1469-185X.2012.00228.x. 22540874

[araf116-B82] Stynoski JL, Shelton G, Stynoski P. 2014a. Maternally derived chemical defences are an effective deterrent against some predators of poison frog tadpoles (Oophaga pumilio). Biol Lett. 10:20140187–20140187. 10.1098/rsbl.2014.0187. 24850895 PMC4046375

[araf116-B83] Stynoski JL, Torres-Mendoza Y, Sasa-Marin M, Saporito RA. 2014b. Evidence of maternal provisioning of alkaloid-based chemical defenses in the strawberry poison frog Oophaga pumilio. Ecology. 95:587–593. 10.1890/13-0927.1. 24804437

[araf116-B84] Tanaka Y . 1996. Social selection and the evolution of animal signals. Evolution. 50:512–523. 10.1111/j.1558-5646.1996.tb03864.x. 28568955

[araf116-B85] Thorogood R, Kokko H, Mappes J. 2018. Social transmission of avoidance among predators facilitates the spread of novel prey. Nat Ecol Evol. 2:254–261. 10.1038/s41559-017-0418-x. 29255302

[araf116-B86] Trumbo ST . 2007. Defending young biparentally: female risk-taking with and without a male in the burying beetle, Nicrophorus pustulatus. Behav Ecol Sociobiol. 61:1717–1723. 10.1007/s00265-007-0403-5.

[araf116-B87] Van Meyel S, Meunier J. 2022. Costs and benefits of isolation from siblings during family life in adult earwigs. Anim Behav. 193:91–99. 10.1016/j.anbehav.2022.09.003.

[araf116-B88] Wade MJ, Bijma P, Ellen ED, Muir W. 2010. Group selection and social evolution in domesticated animals. Evol Appl. 3:453–465. 10.1111/j.1752-4571.2010.00147.x. 25567938 PMC3352501

[araf116-B89] Wang L, Cornell SJ, Speed MP, Arbuckle K. 2021. Coevolution of group-living and aposematism in caterpillars: warning colouration may facilitate the evolution from group-living to solitary habits. BMC Ecol Evol. 21:25. 10.1186/s12862-020-01738-w. 33583398 PMC7883577

[araf116-B90] West-Eberhard MJ . 1983. Sexual selection, social competition, and speciation. Q Rev Biol. 58:155–183. 10.1086/413215.

[araf116-B91] White TE, Umbers KDL. 2021. Meta-analytic evidence for quantitative honesty in aposematic signals. Proc Biol Sci. 288:20210679. 10.1098/rspb.2021.0679. 33906408 PMC8080005

[araf116-B92] Williams BL, Hanifin CT, Brodie ED, Caldwell RL. 2011. Ontogeny of tetrodotoxin levels in blue-ringed octopuses: maternal investment and apparent independent production in offspring of Hapalochlaena lunulata. J Chem Ecol. 37:10–17. 10.1007/s10886-010-9901-4. 21165679

[araf116-B93] Winters AE, Stevens M, Mitchell C, Blomberg SP, Blount JD. 2014. Maternal effects and warning signal honesty in eggs and offspring of an aposematic ladybird beetle. Funct Ecol. 28:1187–1196. 10.1111/1365-2435.12266.

[araf116-B94] Wolf JB, Brodie ED 3rd, Moore AJ. 1999. Interacting phenotypes and the evolutionary process. II. Selection resulting from social interactions. Am Nat. 153:254–266. 10.1086/303168. 29585974

[araf116-B95] Wormington JD, Luttbeg B. 2018. Red clypeal membrane color predicts immune function in a burying beetle (Coleoptera: Silphidae). J Zool. 304:284–292. 10.1111/jzo.12528.

[araf116-B96] Wormington JD, Luttbeg B. 2019. Disrupting information alters the behavioral response to a mutual signal trait in both sexes of Nicrophorus (Coleoptera: Silphidae) burying beetles. Behav Ecol. 30:960–967. 10.1093/beheco/arz035.

[araf116-B97] Zuur AF, Ieno EN, Elphick CS. 2010. A protocol for data exploration to avoid common statistical problems. Methods Ecol Evol. 1:3–14. 10.1111/j.2041-210X.2009.00001.x.

