# Supplementary material for: Maternal care shapes an aposematic display and provides lifelong protection against predators
Source: Behav Ecol. 2025 Sep 29;36(5):araf116. doi: 10.1093/beheco/araf116 (PMC12541378; doi:10.1093/beheco/araf116)
Supplement: araf116_Supplementary_Data [file araf116_supplementary_data.docx]

**Supplementary information**

# Parental care shapes evolution of aposematism and provides lifelong protection against predators

Lindstedt, C.^1^, Boncoraglio, G. ^2^, Cotter, S.C.^2,3^, Gilbert, J.D.J.^2,4^ and Kilner, R.M^2^

1. Department of Forest Sciences, University of Helsinki, Finland

2. Department of Zoology, University of Cambridge, UK

3. School of Natural Sciences, University of Lincoln, UK, LN4 2UE

4. School of Life and Environmental Sciences, The University of Hull, UK.

**SUPPLEMENTARY FIGURES**


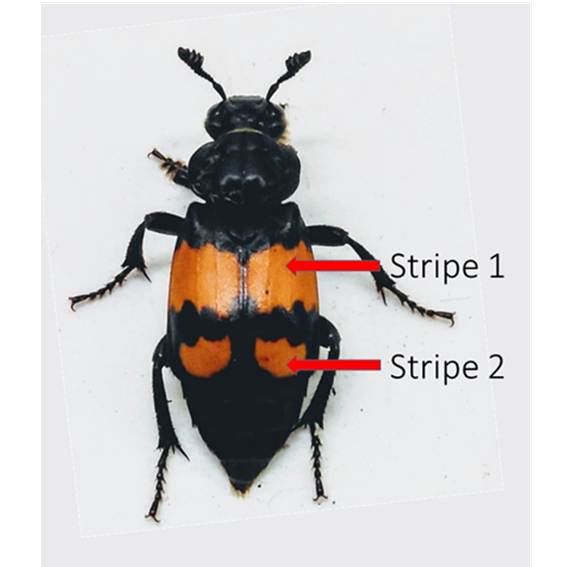


**Figure S1** – A *Nicrophorus vespilloides* adult showing two stripes of conspicuous orange markings on its elytra.

| 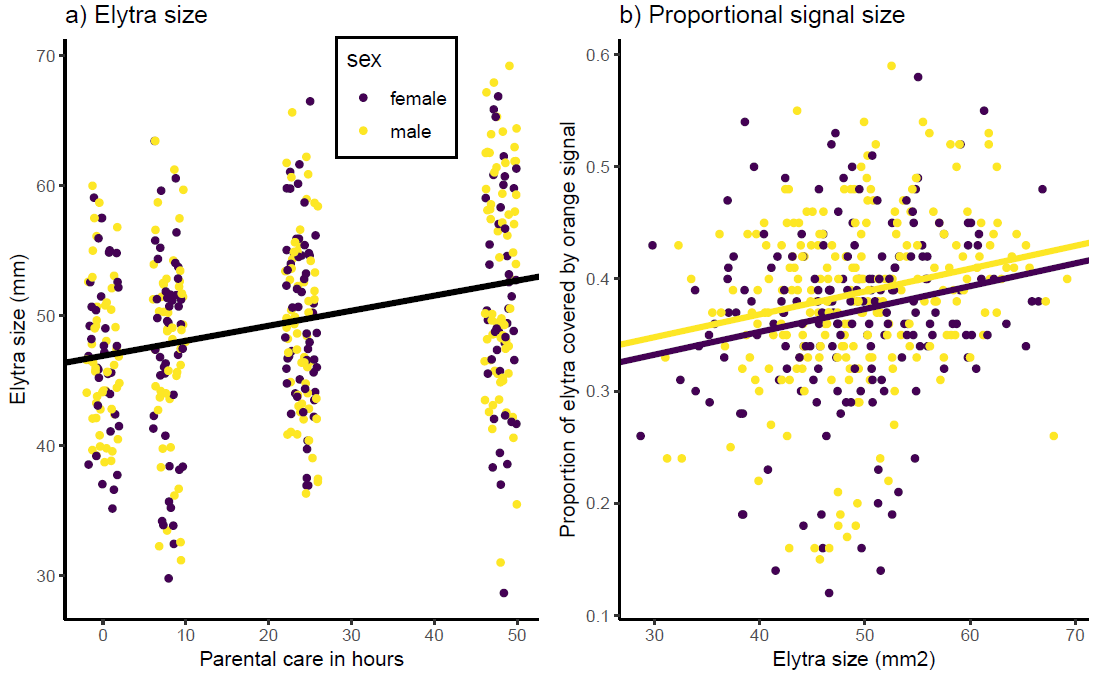 |
| --- |
| **Figure S2** – (a) The change in elytra size by treatment and (b) the relationship between proportional signal size and the size of the elytra, shown for males and females. The fitted lines are predictions from the GLMM. |

| 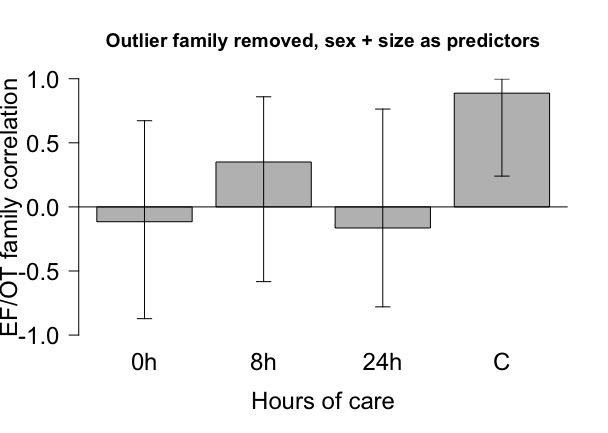 |
| --- |
| 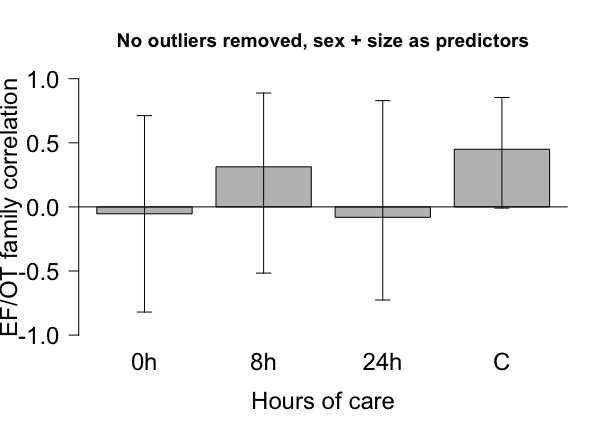 |
| **Figure S3.** A representation of the family level correlation between signal size and the quantity of the defensive fluid produced for No care, 8 hours of care, 24 hours of care and 48 hours of care beetles without and with outlier. The family correlation was estimated using MCMCglmm and was significant for the 48 hours of care treatment only. |

**Table S1. The effects of care duration on body size. Terms retained in the final model are in bold.**

|  | **Body mass in mg** | | | **Elytra length** | | |
| --- | --- | --- | --- | --- | --- | --- |
|  | **F** | **df** | **P** | **F** | **df** | **P** |
| **Care duration** | **17.185** | **1,93.85** | **<0.001** | **8.083** | **1,47.65** | **0.007** |
| **Sex** | **14.501** | **1,737.33** | **<0.001** | 0.267 | 1,373.64 | 0.605 |
| **Care duration:Sex** | 2.025 | 1,735.42 | 0.1552 | 0.428 | 1,372.68 | 0.513 |

**Table S2. The effects of care duration on eclosion fluid volume, repellency and orange signal size. Terms retained in the final model are in bold. For fluid repellency only, the test statistic reported is** $\chi$^2^.

|  |  | **Type 1** | | | **Type 3** | | |
| --- | --- | --- | --- | --- | --- | --- | --- |
|  |  | **F** | **df** | **P** | **F /** $\chi$^2^ | **df** | **P** |
| **Eclosion fluid volume** | **Care duration** | **6.87** | **1,43.69** | **0.012** | 2.85 | 1,46.43 | 0.098 |
|  | **Sex** | **3.91** | **1,382.23** | **0.049** | **4.82** | **1,380.27** | **0.029** |
|  | **Elytra size** | **21.38** | **1,324.29** | **<0.001** | **21.38** | **1,324.29** | **<0.001** |
|  | **Care duration: Sex** | 0.29 | 1,379.73 | 0.588 |  |  |  |
|  | **Care duration: Elytra size** | 1.27 | 1,290.33 | 0.260 |  |  |  |
|  | **Sex: Elytra size** | 0.26 | 1,377.36 | 0.612 |  |  |  |
|  | **Care duration:Sex: Elytra size** | 3.07 | 1,373.15 | 0.081 |  |  |  |
| **Fluid repellencey** | **Care duration** |  |  |  | 2.48 | 1 | 0.115 |
|  | **Sex** |  |  |  | **44.01** | **1** | **<0.001** |
|  | Care duration: Sex |  |  |  | 0.49 | 1 | 0.484 |
|  |  |  |  |  |  |  |  |
|  |  |  |  |  |  |  |  |
|  |  |  |  |  |  |  |  |
|  |  |  |  |  |  |  |  |
| **Orange signal size** | **Care duration** | **8.25** | **1,49.99** | **0.006** | 0.32 | 1,51.24 | 0.575 |
|  | **Sex** | **7.68** | **1,373.12** | **0.006** | **3.86** | **1,372.30** | **0.050** |
|  | **Elytra size** | **430.11** | **1,406.99** | **<0.001** | **430.11** | **1,406.99** | **<0.001** |
|  | **Care duration:Sex** | 1.08 | 1,370.54 | 0.299 |  |  |  |
|  | **Care duration: Elytra size** | 1.95 | 1,408.48 | 0.163 |  |  |  |
|  | **Sex:Elytra size** | 2.10 | 1,369.16 | 0.148 |  |  |  |
|  | **Care duration:Sex:Elytra size** | 2.83 | 1,366.18 | 0.093 |  |  |  |

**Table S3. The effects of care duration on the brightness and saturation of the orange signal. Terms retained in the final model are in bold**

|  |  | **Type 1** | | | **Type 3** | | |
| --- | --- | --- | --- | --- | --- | --- | --- |
|  |  | **F** | **df** | **P** | **F** | **df** | **P** |
| **Brightness Stripe 1** | **Care duration** | **4.94** | **1, 42.34** | **0.032** | **4.94** | **1 42.34** | **0.032** |
|  | **Sex** | 0.71 | 1,342.74 | 0.399 | 0.71 | 1 342.74 | 0.399 |
|  | **Elytra size** | 2.59 | 1,268.47 | 0.110 |  |  |  |
|  | **Care duration:Sex** | 3.23 | 1,340.74 | 0.073 |  |  |  |
|  | **Care duration: Elytra size** | 2.10 | 1,224.90 | 0.149 |  |  |  |
|  | **Sex:Elytra size** | 1.80 | 1,343.67 | 0.181 |  |  |  |
|  | **Care duration:Sex:Elytra size** | 0.00 | 1,339.81 | 0.964 |  |  |  |
| **Brightness Stripe 2** | **Care duration** | 1.47 | 1,41.13 | 0.232 |  |  |  |
|  | **Sex** | 1.13 | 1,338.20 | 0.289 |  |  |  |
|  | **Elytra size** | **4.63** | **1 340.55** | **0.032** | **4.63** | **1 340.55** | **0.032** |
|  | **Care duration:Sex** | 0.00 | 1,334.20 | 0.987 |  |  |  |
|  | **Care duration: Elytra size** | 0.01 | 1,312.03 | 0.921 |  |  |  |
|  | **Sex:Elytra size** | 1.84 | 1,338.56 | 0.176 |  |  |  |
|  | **Care duration:Sex:Elytra size** | 0.00 | 1,334.53 | 0.963 |  |  |  |
| **Saturation Stripe 1** | **Care duration** | 0.05 | 1 42.56 | 0.819 | 0.82 | 1 45.56 | 0.370 |
|  | **Sex** | **5.25** | **1 346.44** | **0.023** | **5.29** | **1 346.51** | **0.022** |
|  | **Elytra size** | **7.24** | **1 172.95** | **0.008** | **7.24** | **1 172.95** | **0.008** |
|  | **Care duration:Sex** | 2.55 | 1 343.70 | 0.111 |  |  |  |
|  | **Care duration: Elytra size** | 0.00 | 1 167.36 | 0.959 |  |  |  |
|  | **Sex:Elytra size** | 0.12 | 1 349.98 | 0.728 |  |  |  |
|  | **Care duration:Sex:Elytra size** | 0.66 | 1 345.43 | 0.417 |  |  |  |
| **Saturation Stripe 2** | **Care duration** | **6.41** | **1,40.84** | **0.015** | **0.60** | **1 43.48** | **0.442** |
|  | **Sex** | **12.39** | **1,341.12** | **<0.001** | **15.97** | **1 343.98** | **<0.001** |
|  | **Elytra size** | **87.01** | **1,281.36** | **<0.001** | **87.75** | **1 281.33** | **<0.001** |
|  | **Care duration:Sex** | **4.90** | **1,339.66** | **0.028** | **4.90** | **1 339.66** | **0.028** |
|  | **Care duration: Elytra size** | 1.58 | 1,245.29 | 0.211 |  |  |  |
|  | **Sex:Elytra size** | 0.82 | 1,341.74 | 0.365 |  |  |  |
|  | **Care duration:Sex:Elytra size** | 1.07 | 1,337.95 | 0.301 |  |  |  |
| **Brightness Black** | **Care duration** | 2.36 | 1,10.22 | 0.155 |  |  |  |
|  | **Sex** | 2.71 | 1,13.34 | 0.123 |  |  |  |
|  | **Elytra size** | 0.01 | 1,12.69 | 0.927 |  |  |  |
|  | **Care duration:Sex** | 0.07 | 1,13.19 | 0.803 |  |  |  |
|  | **Care duration: Elytra size** | 3.38 | 1,12.47 | 0.090 |  |  |  |
|  | **Sex:Elytra size** | 2.05 | 1,13.18 | 0.175 |  |  |  |
|  | **Care duration:Sex:Elytra size** | 0.13 | 1,13.17 | 0.728 |  |  |  |
| **Saturation Black** | **Care duration** | 0.09 | 1,41.22 | 0.767 | 0.20 | 1,41.73 | 0.655 |
|  | **Sex** | **3.44** | **1,345.77** | **0.064** | **7.24** | **1,348.92** | **0.007** |
|  | **Elytra size** | 1.08 | 1,185.59 | 0.300 |  |  |  |
|  | **Care duration:Sex** | **3.84** | **1,344.28** | **0.051** | **3.84** | **1,344.28** | **0.051** |
|  | **Care duration: Elytra size** | 0.55 | 1,162.33 | 0.460 |  |  |  |
|  | **Sex:Elytra size** | 0.77 | 1,349.23 | 0.382 |  |  |  |
|  | **Care duration:Sex:Elytra size** | 0.11 | 1, 344.44 | 0.741 |  |  |  |
